# Comparison of Effectiveness of Two Different Practical Approaches to Teaching Basic Life Support and Use of an Automated External Defibrillator in Primary School Children

**DOI:** 10.3390/medicina60081363

**Published:** 2024-08-21

**Authors:** Nadja Pitz Durič, Vesna Borovnik Lesjak, Matej Strnad

**Affiliations:** 1 Emergency Medicine Department, Faculty of Medicine, University of Maribor, Taborska ul. 8, 2000 Maribor, Slovenia; nadja.pitz@student.um.si (N.P.D.); strnad.matej78@gmail.com (M.S.); 2 Prehospital Unit, Emergency Medical Services Unit, Community Health Center Dr Adolfa Drolca Maribor, Cesta Proletarskih Brigad 21, 2000 Maribor, Slovenia; 3 Emergency Department, University Medical Center Maribor, Ljubljanska ul 5, 2000 Maribor, Slovenia

**Keywords:** automatic external defibrillator, cardiopulmonary resuscitation, out-of-hospital cardiac arrest, schoolchildren, teaching

## Abstract

*Background and Objectives*: As the first three links of the chain of survival of victims of cardiac arrest depend on prompt action by bystanders, it is important to educate as much of the population as possible about basic life support and use of an automatic external defibrillator (BLS and AED). Schoolchildren are an accessible population that can be easily taught and numerous BLS and AED courses are available. The aim of this study was to assess the effectiveness of two different practical approaches to teaching BLS and AED. *Material and Methods*: We compared two different BLS and AED courses (course A and B) offered to 280 eighth- and ninth-grade students in primary schools. Knowledge about and the intention to perform BLS and AED were evaluated using validated questionnaires before and after the courses. Descriptive methods were used to describe the results. To compare courses, we used the Mann–Whitney U test. A *p* value of <0.05 was considered statistically significant. *Results*: Differences in knowledge and intention to perform BLS and AED after the courses were significant between courses (*p* < 0.001 and *p* = 0.037, respectively). After course A, students demonstrated significantly better knowledge and numerically greater intention to perform BLS and AED (intention score 6.55 ± 0.61 out of 7). *Conclusions*: Courses in which students have the opportunity to individually practice BLS skills show a greater increase in knowledge and in intention to perform BLS and AED.

## 1. Introduction

Sudden cardiac arrest is the sudden cessation of cardiac function, with hemodynamic collapse. Patients are unresponsive, not breathing and show no signs of circulatory function. Without the performance of basic life support (BLS) and the use of an automated external defibrillator (AED), sudden cardiac death rapidly occurs [1].

Out-of-hospital cardiac arrest (OHCA) is the third leading cause of death in Europe [2]. The proportion of patients who experience OHCA in whom resuscitation is initiated by bystanders varies widely between (and even within) countries, averaging 58% (range 13% to 82%) [3]. AED use is still rare in Europe, with an average of 28% (range 3.8–59%) [4]. Survival to hospital discharge is on average 8%, ranging from 0 to 18% [3].

Survival after cardiac arrest depends on a sequence of actions, known as the chain of survival. The latter is based on four links: (1) early recognition of cardiac arrest and call for help; (2) early initiation of BLS; (3) early use of an AED; and (4) post-resuscitation medical care [5]. Because of the importance of early intervention, it is important that the largest possible percentage of the public is educated about BLS and AED care [6].

Systematic teaching of schoolchildren about BLS and AED has been shown to be an effective way of encouraging active participation with BLS implementation by lay people in cases of OHCA [7]. Teaching schoolchildren is easy and cost-effective, especially if taught as part of compulsory school activities, and is therefore promoted worldwide [8,9]. Of course, effective implementation of BLS and AED teaching depends greatly on their age and physical disposition [10,11]. 

In 2015, the World Health Organization supported the global initiative “Kids save Lives”, which advocates the inclusion of BLS courses in school curricula for students from 12 years of age [9]. It has been found that school-age children are motivated, learn faster, are aware of the importance of helping others and are willing to learn BLS [12]. Educating children also creates a population that could pass on the knowledge to family members and friends and potentially later on as instructors to the general lay public. 

Courses not only enhance theoretical knowledge of resuscitation but also promote prosocial behavior in children [13]. Potentially, their attitudes towards resuscitation may change as they gain the confidence to perform BLS, become self-efficient, increase their intention to help others and improve empathy [14,15]. 

In Slovenia, BLS courses are not yet included in the regular curriculum, so the implementation of courses depends on the interest shown by individual schools. At a national level, training on BLS and AED use is offered by the Red Cross [16] and the National Institute of Public Health through its Health Education activity [17]. Locally, training is offered by various associations and projects within health centers.

The way in which BLS and AED courses are delivered is currently up to the individual provider. The most recent resuscitation guidelines include recommendations that training should be tailored to the target population [4]. Annual short refresher courses are recommended, as well as the use of advanced technologies for learning (mobile phones, apps, games) [18,19,20,21]. Virtual reality could also be used in the future, but further development will be needed to allow the experience of appropriate depth of chest compressions and, in general, the skills that trainees acquire in traditional resuscitation courses [22]. However, a growing number of studies demonstrate the importance of a hands-on approach to learning BLS, which is also emphasized in the latest resuscitation guidelines [19]. 

The impact of courses on the improvement of theoretical knowledge is immediate [14,15,23,24,25]. The effectiveness of the courses, however, is demonstrated in practice by behavior when faced with the actual need for resuscitation. As this behavior cannot be directly measured, the measurement of intention to perform BLS serves as a surrogate for behavior [26]. 

Our study aimed to determine whether different modes of delivering BLS and AED courses have different impacts on the effectiveness of the course. To measure this, we used validated instruments to assess the effectiveness of two different BLS and AED courses that are currently being delivered to primary school children in the region. Based on the results, we analyzed the impact of different instructor backgrounds and practical approaches on the overall effectiveness of the courses, that is, on participants’ knowledge and intention to actually perform BLS and AED in a real-life event.

## 2. Materials and Methods 

### 2.1. Description of Courses

Two courses were compared in the study, organized by two different institutions with different course formats. The characteristics and differences in the two courses are described in Table 1. Courses were designed and conducted for individual classes of students (there are up to 30 students per class). Course A involved three or two instructors, depending on the number of students in a class (up to 20 students—two instructors; up to 30 students—three instructors). There were 7 groups of students (e.g., two classes with 16 and two with 25 students, and classes with 19, 20, 26 and 30 students each). Course B involved two or four instructors per class (there were two classes with 20 and 17 students each and two with 29 students, respectively). Instructors in course A were professional emergency medical service team members, whereas in course B, the instructors were medical students. All instructors used the same computer screen presentation for the theoretical lecture that took place in either the home classroom or the gym (physical education room). Skills practice took place in the gym and consisted first of a practical demonstration with discussion of BLS (including rescue breaths) for the whole class followed by individual performance by each student on the torso manikins (Little Anne, Laerdal Medical, Stavanger, Norway). Students individually chose whether they were willing to practice rescue breaths or not (students wiped their own manikin before use and used a specific manikin mouth shield). Instructors provided verbal feedback during the training. Content of the courses was compliant with the European Resuscitation Council guidelines valid at the time that do not differ substantially in regard to BLS measures [27,28]. 

### 2.2. Sample

The study population were 8th- and 9th-grade students in primary schools. The included schools decided to partake in the offered course for the first time. The average age of students was 13 and 14, respectively. Sampling took place from November to December 2018 for course A and in March 2023 for course B. 

### 2.3. Measuring Instrument

A validated questionnaire was used (Supplementary Materials File S1) [26,29]. The questionnaires were distributed to and anonymously fulfilled by the students as a paper and pen test before and immediately after the course. Only questionnaires on which the students indicated that they agreed to filling out the questionnaire and the use of the data were used. Four students in course A and twenty-five in course B did not agree or did not fill out the agreement statement and were therefore not included in the study sample.

The questionnaire consisted of three parts:

(a) The first part included sociodemographic data: gender, grade and any previous participation in any kind of BLS and AED course (not only school courses).

(b) The second part was a knowledge test. It consisted of 10 questions on BLS and AED with five possible answers. The wording was adapted to the understanding of the tested population. Out of the five possible answers, only one was correct, which means that the maximum possible score was 10. The fifth answer always offered the option “I don’t know”, which was used to avoid guessing and was considered incorrect in the subsequent analysis.

(c) The third part was a questionnaire on the intention to perform BLS and AED. It included 4 statements with 7 possible levels of agreement on a Likert scale ranging from “strongly disagree” (1) to “strongly agree” (7) and with an intermediate neutral level (4). The minimum possible score was 4 and the maximum possible score was 28. Included statements were as follows: “If I were the only witness when someone collapsed, I would help”; “If I were the only witness to a cardiac arrest in an unknown person, I would start resuscitating”; “If I were the only witness to a cardiac arrest in a known person (friend, family member), I would start resuscitating”; and “If I had an AED available, I would use it (on a known or unknown person in cardiac arrest)”.

Permission to conduct the study was requested from and granted by the National Medical Ethics Committee (23 October 2018, number 0120-549/2017/9). As per Slovenian rules for such type of educational research, general written consent of the parents was collected in advance at the beginning of each school year.

### 2.4. Statistical Analysis

Descriptive methods were used to present the results of the knowledge and intention questionnaires. Scores on the knowledge test were presented as mean total scores (with standard deviations) ranging from 1 to 10. Scores on the intention part were presented as mean total scores (with standard deviations) ranging from 1 to 7. A paired samples T-test was used to compare means before and after each course. The Mann–Whitney U test was used to compare the results of the two courses. A two-sided *p* value < 0.05 was considered statistically significant. IBM SPSS Statistics software (version 28; SPSS Inc., Chicago, IL, USA) was used for statistical data analysis.

## 3. Results

Demographic characteristics of the sample are presented in Table 2.

The percentages of correct answers on the individual questions on the knowledge test are presented in Table 3. 

**Figure 1:**
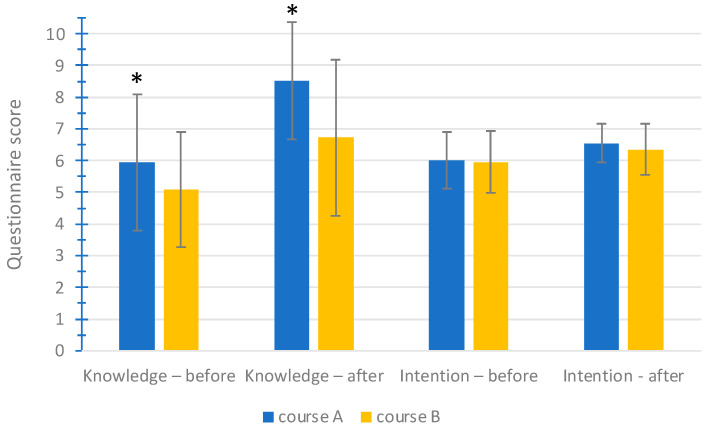
Knowledge and intention before and after the courses. Presented are mean values and standard deviations. Notes: *, *p* < 0.001.

The results of the cumulative knowledge test and the intention questionnaire before and after the course are presented in Figure 1. There were statistically significant differences in baseline levels of knowledge between students before attending the two courses.

Differences in knowledge immediately after the courses were also significant (8.52 and 6.73 for course A and B, respectively). There were no significant differences in pre-course intention between students from different schools (6.01 and 5.95 for course A and B, respectively). There was a significant increase in intention after each individual course (*p* < 0.05). Students after course A showed a numerically higher level of intention compared to students after course B (0.21 higher), but the difference did not reach statistical significance. There was a greater net increase in knowledge and intention after course A compared to course B (*p* = 0.022 for knowledge and *p* = 0.037 for intention, respectively).

Additionally, we analyzed potential differences in knowledge and intention between girls and boys and those who had attended any previous BLS courses and those who had not (Table 4). The only significant difference was in baseline knowledge where girls scored better on the knowledge test before the course. Attendance to any previous course made no difference in knowledge or intention.

## 4. Discussion

In our efforts to empower children to be willing to perform BLS and AED, it is necessary to emphasize the quality of the teaching and courses. These need to be tailored to the target population and the most effective way of teaching needs to be determined [4]. Improving knowledge alone is not enough to conclude that a course is effective. More important is the impact of courses on learners’ intention to put this knowledge into practice in a situation where they witness a sudden cardiac arrest, that is, to actually start BLS and use an AED. On one hand, we need to encourage children to use the knowledge about BLS and AED they have acquired in a course in a real-life situation. On the other hand, it is necessary to provide an effective course that can be easily implemented in busy school curricula to ensure systematic and continuous learning for primary school children [7,9,30]. In the context of our study, two different courses were compared with each other using validated questionnaires to determine the most effective way of teaching BLS and AED to schoolchildren.

Significant differences were observed between the two different groups of students in their baseline knowledge of BLS and AED before the courses. The most important difference (and at the same time—a limitation) between the populations of students, was the timing of the courses. Course A was delivered before the SARS-CoV-2 pandemic, while course B was delivered after the pandemic. During the pandemic, children changed their lifestyle habits, they had unequal opportunities for distance education, many developed mental health problems and aggressive behavior patterns, their sleeping habits and amount of sleep changed and they spent more time in front of screens [31,32,33]. All of these could potentially have affected their learning abilities and achievements and could explain the poorer pre-course knowledge of BLS and AED. Another possible factor affecting the level of knowledge is younger age—course B included eighth- and ninth-grade students, whereas course A only included ninth graders. 

The level of knowledge of both groups of students improved after the courses, which was in accordance with previous reports by several authors [14,34,35,36]. Knowledge gain was more prominent after course A compared to course B. In course A, each student practiced on his/her own manikin. The latter allows the students to practice their BLS and AED skills repeatedly and uninterruptedly. Repetition of learned knowledge has been shown to improve performance [37]. Existing studies have already demonstrated the importance of hands-on training and repetition of BLS steps [13,23,26,29]. In course B, where the whole class shared two manikins, the level of knowledge was lower (6.73 vs. 8.52). 

The most important predictor of behavior in an actual sudden cardiac arrest event (i.e., performance of BLS and AED), however, is the intention of the children to perform the resuscitation. Encouragingly, both groups of children expressed a favorable baseline intention to perform BLS and AED even before the course (all above the score of 5 out of 7 on a Likert scale) and there were no statistically significant differences between groups before the course. This is consistent with previous findings that school-aged children are motivated and aware of the importance of helping others [12]. 

More important than the baseline level of knowledge and intention is the intention to perform BLS and AED after a course, as this serves as an indicator of the attitude towards resuscitation that the child has adopted after a course. There were no significant differences in intention to perform BLS and AED after the course between children in different courses. Intention after course A was only a few percentage points higher than after course B. In course A, each student practiced on his/her own manikin and had enough time to repeat the practical skills several times in 45 min. In course B, only two manikins were available for the whole group of students but a larger number of instructors were available, potentially allowing for more interaction among students and instructors.

Courses also differed in the professional background of the instructors, all of whom came from a medical background. We did not expect a significant effect of instructor background on course performance in terms of knowledge improvement, as several studies have so far shown that professional background of the BLS instructor does not play a vital role in knowledge gain as health professionals, teachers and medical students are equally successful in improving knowledge [13,34,36,38,39,40]. However, there have been no previous reports on the effect of professional background on intention to date. The results of our study suggest that medical students are also not inferior to health professionals (paramedics/physicians) in strengthening schoolchildren’s intention to actually perform BLS and AED.

Interestingly, there were no differences in knowledge and intention based on previous attendance to any BLS and AED course among students. We also checked for differences among boys and girls and found no meaningful differences. There were only differences in baseline knowledge where girls showed better knowledge. Previous reports on the effect of gender or of having beforehand completed a BLS training course on knowledge and intention are inconclusive on the matter [41,42]. This potentially highlights the need to put more emphasis on the development and implementation of high-quality and population-tailored courses.

We found that both knowledge and intention scores were numerically higher in students who attended courses where everyone had the opportunity to practice on their own manikin and had enough time to perform several repetitions of the practices. Based on the results, we suggest that BLS and AED courses primarily focus on quality practical approaches to teaching with sufficient hands-on time under the supervision of a qualified instructor. Thereby, the time and resources needed to implement BLS and AED courses into school curricula might be optimized. However, due to several limitations of our study, further larger-scale comparative studies are warranted to confirm our findings.

### Limitations

Courses were led by different instructors. Despite the use of the same teaching aids for the trainees, the teaching also involves unpredictable and uncontrollable factors beyond our control, such as different instructors’ characters, communication skills, instructors’ commitment and trainees’ current mood. Also, the courses were delivered at different times and with different school timetables for the children, which could affect their concentration and willingness to participate.

A possible limitation could be the fact that the courses were held in different time periods. Most of the courses were conducted before the SARS-CoV-2 pandemic, whereas one course was conducted after the pandemic, which could have influenced the test results for the reasons mentioned above. The temporal distribution of included courses also yielded nonequal characteristics (different sample size, different student age) of the sample determined by convenience as the study was limited with resources and time. The original design of the study intended to include both courses in the same time frame but was unfortunately interrupted by the COVID pandemic.

The primary schools included were from different townships in the eastern part of Slovenia, which, given the geographically small size of Slovenia, should not be a factor influencing children’s prior knowledge or intention to perform BLS and AED.

Another limitation of this study is that only short-term knowledge and intention were assessed. Studies of long-term retention of knowledge and intention and assessment of practical skills are necessary to make more concrete and specific recommendations for future courses.

## 5. Conclusions

Across both courses, the students’ level of knowledge has improved as expected. The best knowledge was demonstrated by the students who practiced on their own manikins. The level of intention also increased after both courses. In light of this, we find that the effectiveness of a course depends on the opportunity of students to practice on manikins under close supervision of a qualified instructor. 

## Figures and Tables

**Table 1:** Characteristics of each course.

	Course A	Course B
City/Town	Maribor	Lovrenc na Pohorju, Miklavž na Dravskem polju
Organizer	EMS Health Center Maribor	Association of Medical Students Maribor
Instructor	Physician, registered nurse/paramedic	3rd and 2nd year medical students
Number of instructors	1 per 10 students	1 per 7 to 10 students
Course format		
Theoretical lecture	45 min	45 min
Skills practice	45 min	45 min
Number of manikins	1 per student	2 per class
Class	9th	8th and 9th

Legend: EMS, emergency medical services.

**Table 2:** Demographic characteristics of the sample.

Course	Number of Students	Boys	Girls	8th Grade	9th Grade	Previous Course—Yes
A	173	94	79	/	173	25
B	107	48	59	79	28	20

**Table 3:** Percentages of correct answers on the knowledge test.

	Question	% of Correct Answers before		% of Correct Answers after	
		Course A	Course B	Course A	Course B
1	How do you recognize a person in cardiac arrest?	73%	56%	78%	61%
2	Who can help in a case of cardiac arrest?	78%	88%	94%	85%
3	A person suddenly loses consciousness and collapses. What do you do?	64%	63%	89%	83%
4	How do you check if a person is breathing normally?	83%	77%	90%	87%
5	What kind of breathing is NOT a sign of life?	64%	1%	88%	1%
6	How is basic life support correctly performed?	44%	46%	93%	89%
7	On the sketch of the torso below, mark with a cross the correct site for chest compressions during basic life support:	53%	40%	71%	65%
8	How do you perform artificial breaths in an unconscious person?	61%	54%	91%	81%
9	What do you do if you are unsure whether a person is in cardiac arrest or not?	34%	27%	73%	40%
10	What is an AED (automatic external defibrillator)?	42%	57%	86%	81%

**Table 4:** Comparison of knowledge and intention scores between genders and previous training.

	Knowledge before	Knowledge after	Intention before	Intention after
Girls	5.35 (2.08) *	7.63 (2.28)	6.05 (0.83)	6.40 (0.72)
Boys	5.91 (2.02)	8.05 (2.26)	5.93 (1.02)	6.55 (0.66)
Previous course	6.16 (2.09)	7.67 (2.82)	6.16 (0.873)	6.63 (0.5)
No previous course	5.68 (1.97)	8.02 (1.94)	5.96 (0.93)	6.44 (0.74)

Legend: presented are means and standard deviations. *, *p* < 0.05.

## Data Availability

Data are available upon request from the corresponding author.

## Supplementary Materials

The following supporting information can be downloaded at https://www.mdpi.com/1648-9144/60/8/1363/s1, File S1: Questionnaire on knowledge and intention.
