# Supplementary material for: Comparison of Effectiveness of Two Different Practical Approaches to Teaching Basic Life Support and Use of an Automated External Defibrillator in Primary School Children
Source: Medicina (Kaunas). 2024 Aug 21;60(8):1363. doi: 10.3390/medicina60081363 (PMC11487411; doi:10.3390/medicina60081363)
Supplement: Supplementary file 1 [file medicina-60-01363-s001.zip › medicina-3106854-supplementary.pdf]

Knowledge and teaching of basic life support with the use of automated external defibrillator and attitude towards resuscitation in primary school children

Here is a questionnaire with the help of which we will try to determine your current knowledge of basic life support and how interested you are in the topic. The aim of this study is to improve the efficacy of this training and subsequently the efficacy of resucitation. Participation is voluntary and anonymous. Please pick a code that you will use on both the before- and after-questionnaire and enter it in the square below. Data will be processed and handled in a way that will not allow individual identification. Your cooperation is greatly appreciated.

I agree with fulfilling this questionnaire (circle): Yes No

Code (fill in the square by your own choice):

Class (circle): 7. 8. 9.

Sex (circle): M F

Have you received prior training in basic life support? Yes No

A. ***With the following quiestions we want to test your basic life support (BLS) knowledge. Circle a single most fitting answer.***

1. How do you recognize a person in cardiac arrest?
   a. The person shrieks loudly and is in pain.
   b. The person is unconscious, but breathes normally.
   c. The person is conscious and breathes deeply.
   d. The person does not show any signs of life.
   e. I don't know.

2. Who can help in a case of cardiac arrest?
   a. Only cardiac surgeons.
   b. Only health professionals.
   c. Only those with a course in first aid.
   d. Anybody with appropriate knowledge.
   e. I don't know.

3. A person suddenly loses consciousness and collapses. What do you do?
   a. I shake the person vigorously to try to awaken him/her and then wait for help.
   b. I gently shake the person to check responsivness, then I check breathing, and then I call for help.
   c. I don't touch the person and call for help.
   d. I immediately start chest compressions and rescue breathing.
   e. I don't know.

4. How do you check if a person is breathing **normally**?
    a. If I hear only irregular, occassional, loud breaths, I assume the person is breathing normally.
    b. To check for normal breathing, I must first relieve the airway, then look, feel, and listen for normal breathing.
    c. Until the person doesn't look weird or blue, I assume he/she is breathing normally.
    d. It is enough to evaluate by the way if the chest is rising.
    e. I don't know.

5. What kind of breathing is NOT a sign of life?

    a. Deep breathing in a conscious person.
    b. Fast breathing in a conscious person.
    c. Irregular, beep sighs in an unconscious person.
    d. Regular breathing in an unconscious person.
    e. I don't know.

6. How is basic life support correctly performed?
    f. 15 chest compressions, followed by 1 artificial breath.
    g. 20 chest compressions, followed by 2 artificial breaths.
    h. 30 chest compressions, followed by 2 artificial breaths.
    i. 40 chest compressions, followed by 4 artificial breaths.
    j. I don't know.

7. On the sketch of the torso below mark with a cross the correct site for chest compressions during basic life support:

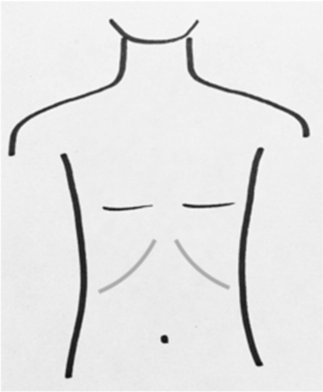

8. How do you perform artificial breaths in an unconscious person?
    a. I give 2 breaths to the mouth or nose until the chest rises.
    b. I quickly blow into the mouth or nose until I am out of breath.
    c. As this is not included in the basic life support I will not do it.
    d. I forcefully blow into the mouth or nose, similar to inflating a balloon.
    e. I don't know.

9. What do you do if you are unsure whether a person is in cardiac arrest or not?
   a. If in doubt, I start basic life support nevertheless.
   b. I place the person in a stable side position and wait for help to arrive.
   c. I wait 5 minutes and then shake the person again.
   d. I wait for the emergency service personnel to arrive and let them decide.
   e. I don't know.

10. What is an AED (automatic external defibrillator)?
   a. An electronic device used by doctors, nurses, and paramedics in cases of cardiac arrest during surgery.
   b. An electronic device that can potentially restart the heartbeat and can be used by anyone.
   c. An electronic devic that can be used by paramedics in cases of cardiac arrest.
   d. An electronic device that can only be used by people with special trainig for AED use.
   e. I don't know.

B. The following questions refer to your attitude towards basic life support (BLS). Circle the number of the answer that best describes your agreement with each statement on the scale from 1 – do not agree at all, 4 – neutral (neither agree nor disagree), and 7 – completely agree.

**Question**	**Do not agree at all**			**Neutral**			**Completely agree**
11. If I were the only witness when someone collapsed I would help.	1	2	3	4	5	6	7
12. If I were the only witness to a cardiac arrest in an unknown person, I would start resuscitating.	1	2	3	4	5	6	7
13. If I were the only witness to a cardiac arrest in a known person (friend, family member), I would start resuscitating.	1	2	3	4	5	6	7
14. If I had an AED available, I would use it (on a known or unknown person in cardiac arrest).	1	2	3	4	5	6	7

Thank you for participating!
